# Reconstructive challenge of dermatofibrosarcoma protuberans in the female breast

**DOI:** 10.1186/1477-7819-9-1

**Published:** 2011-01-07

**Authors:** Tae Hwan Park, Sang Won Seo, June Kyu Kim, Choong Hyun Chang

**Affiliations:** 1 Department of Plastic and Reconstructive Surgery, Kangbuk Samsung Hospital, Sungkyunkwan University School of Medicine, Seoul, Korea

## Abstract

Dermatofibrosarcoma protuberans is an uncommon locally aggressive malignant neoplasm that most frequently appears in the trunk, followed by the extremities, head, and neck. But occurrence in the breast is extremely rare. We present a case of a 28-year-old woman, who had a history of trauma 5 years previously and excision 1 year before presentation at our clinic. We performed wide excision, together with microscopic and immunohistochemical analysis. No postoperative oncologic treatment was used and she remains disease-free 1 year after the surgery without any tumor recurrence. Here, we report a case of dermatofibrosarcoma protuberans in the female breast and present a detailed discussion of the diagnosis and treatment with reference to available literatures.

## Background

Dermatofibrosarcoma protuberans (DFSP) is an uncommon, slow-growing, low-grade sarcoma of putative dermal fibroblastic origin[1]. DFSP was first described by Darier and Ferrand in 1924 and was referred to as a progressive and recurrent dermatofibroma. It was later officially termed "dermatofibrosarcoma protuberans" by Hoffmann in 1925[2].

DFSP is a clinically challenging neoplasm because it is characterized by a high recurrence rate with a high propensity for local invasion. In addition, it is difficult to make a correct preoperative diagnosis because DFSP is often left untreated for several years, probably due to its benign appearance and initial indolent behaviour.

Our case highlights two specific features. First, understanding the natural history of this entity is crucial even though there are potential difficulties in the diagnosis of DFSP owing to its rarity. Second, reconstruction for aesthetic purpose can be delayed since the presence of a flap after immediate reconstruction may prevent detection of the local recurrence and better aesthetic outcomes can be achieved with delayed procedure.

## Case presentation

**Figure 1:**
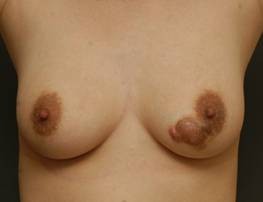
**Preoperative appearance of the patient**. A 2 × 1 cm reddish mass with 3 × 2 cm brownish plaque-like cutaneous thickening.

A 28-year-old woman presented with a slow growing pigmented protruding mass in the medial lower quadrant of her left breast. She had a history of trauma 5-years previously, after which, the mass rapidly grew bigger and became more pigmented. Aware of these changes, she underwent local excision of the mass in another hospital a year before presentation in our clinic and was told that the mass was a benign tumor without being provided with a specific diagnosis. There were no abnormal laboratory findings and her family history and medical history were unremarkable except for the lesion that was excised a year ago. On gross inspection and palpation, we noticed a 2 cm × 1 cm smooth mobile keloid-like reddish mass surrounded by a 3 cm × 2 cm brownish plaque-like cutaneous thickening in the medial lower quadrant, at the 7 o'clock position of her left breast (Figure 1). On physical examination, the lesion was not tender and no regional lymph nodes were palpated. Since the clinical findings were suggestive of benign breast disease and the patient refused further evaluation, we did not perform conventional mammography or ultrasonography.

We performed a wide excision of the breast lesion with a 3 cm margin and a frozen section under general anesthesia and the defect was closed primarily.

**Figure 2:**
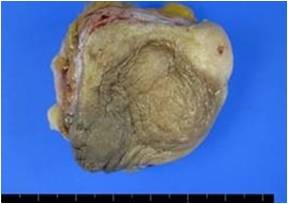
**Gross specimen**. Outer surface of ill-defined gray rubbery mass.

**Figure 3:**
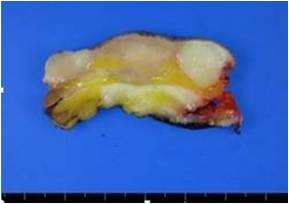
**Gross specimen**. Cross section of the mass.

The gross pathology of the specimens showed a grayish white ill-demarcated soft tissue mass measuring 3.8 cm × 3.1 cm × 1.1 cm (Figure 2, 3).

**Figure 4:**
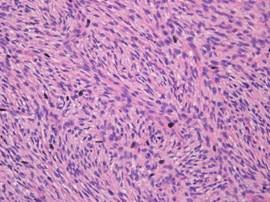
**Microscopic examination**. Several atypical cell with mitosis in the relatively blend spindle cells was noticed (H&E stain, × 400).

**Figure 5:**
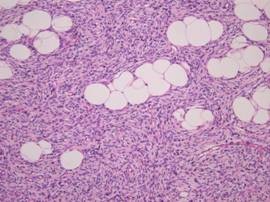
**Microscopic examination**. There is characteristic infiltration of fibroblast between fat cells in subcutaneous tissue (H&E stain, × 200).

**Figure 6:**
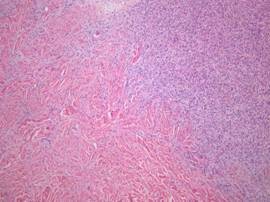
**Microscopic examination**. Infiltrative border in dermis was observed (H&E stain, × 100).

On histological examination, a storiform pattern of spindle cells infiltrating into subcutaneous tissue above the mammary gland was noticed (Figure 4, 5, 6). There was only mild mitotic activity (2-3 per 10 high-power fields) and no resection margin involvement.

**Figure 7:**
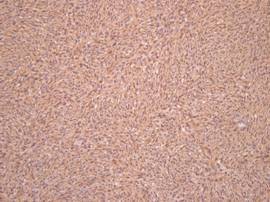
**Immunohistochemical examination**. Tumor stained positive for CD34.

**Figure 8:**
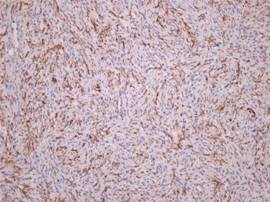
**Immunohistochemical examination**. Tumor stained negative for S-100 protein.

On immunohistochemical examination, the lesion stained positive for CD34 (Figure 7) and locally positive for SMA, and negative for CD68 and S-100 protein (Figure 8). The mass was eventually diagnosed as DFSP. The patient did not undergo any postoperative oncologic treatment. Delayed reconstruction is scheduled for at least 2 years after the wide excision unless recurrence or metastasis occurs. Postoperative follow-up has been maintained up to the present time, 1 year after the operation, without any tumor recurrence.

## Discussion

DFSP accounts for approximately 6% of all soft tissue sarcomas with an estimated incidence of 0.8 cases per million per year [3].

DFSP occurs most frequently between the second to fourth decades of life and males are more frequently affected. Most cases of DFSP present as a plaque-like cutaneous thickening and generally increase in size at a slow rate for a considerable period of time, thus they are often regarded as a benign tumor before more rapid growth ensues[4]. For this reason, it is usually regarded lightly by patients as well as physicians leading to delayed and often 'wait and watch' approach. Differential diagnoses include dermatofibromas, keloid, or morphea in early lesions, or cutaneous metastasis, malignant lymphoma, and Kaposi sarcoma in advanced states [5].

Tohru et al. and Hisaki et el. place a small value on mammography and ultrasonography in preoperative diagnosis of DFSP in the breast[6]. MRI can give information about the deep tissue involvement, especially in patients with large recurrent tumors, whereas, CT has limited value except defining bone involvement. Fine-needle aspiration(FNA) may be useful in establishing a nonspecific pathology in patients with previously treated tumors[7]. However, FNA is not always feasible because obtaining sufficient tissue is difficult for most untreated tumors. Large core needle biopsy or excisional biopsy can help to provide a correct diagnosis[3]. The characteristic findings are interlacing spindle-shaped tumor cells in the dermis and subcutaneous fat layer that form definite bands that interweave or radiate like spokes of a wheel, forming a so-called 'cartwheel' or 'storiform' pattern.

Immunohistochemical markers are highly sensitive for DFSP. In particular, CD34 is a useful marker that allows differentiation of DFSP tumor cells from normal stroma cells. DFSP generally stains positive for CD34 and negative for S-100 protein, factor XIIIa[8].

Many authors share the same opinion that complete surgical excision with wide, pathologically negative margins is the optimal treatment for primary or recurrent DFSP. Traditionally, the recommended treatment has been wide surgical excision with gross margins of at least 3 cm [4,9,10]. In 1967, McPeak *et al *concluded that even with this margin the recurrence rate was 10% (8 of 82)[10]. As many authors believe that the risk of metastasis is increased in cases with several incompletely excised recurrent tumors,[11] surgeons should focus on complete tumor excision to prevent local recurrence, metastasis, and reoperations. Since wide excision usually causes noteworthy distortion and leaves patients with significant cosmetic problems, especially in the female breast, reconstructive procedure is required in almost every instances. In cases in which wide excision and primary flap reconstructions are performed simultaneously, there should be no doubt as to the adequacy of the excision, because the presence of a flap may prevent detection of the local recurrence. In general, the reconstructive challenge in DFSP such as in the trunk, extremities, head and neck comprises of large tissue defects which need covering when vital structures are exposed. In cases of the breast, however, the challenge lay in being able to create a near-normal-looking breasts with good symmetry and color match.

Mohs microscopic surgery (MMS) has emerged as an alternative approach allowing immediate microscopic examination of the margins. However, MMS requires considerable training, a specialized team, multiple stages[12].

Adjuvant radiation therapy can be administered if margins are positive or very close to the tumor after maximal resection.

Recently, the tyrosine kinase inhibitor Imatinib, was shown to induce regression of advanced DFSP when it is impossible to obtain surgical margins. According to a review of Mizutani et el., imatinib inhibited the growth of metastatic lung lesions in a patient with DFSP [13].

In conclusion, although DFSP in the breast is rare, clinical suspicion is of paramount importance for an accurate diagnosis and vigilant follow-up for the pigmented lesion is necessary.

**Figure 9:**
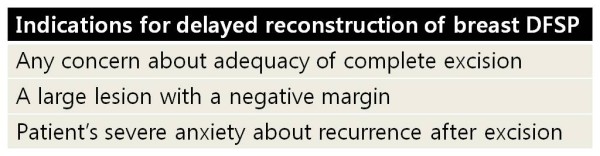
**Indications for delayed reconstruction of breast DFSP**.

Even when the growth pattern of a lesion does have the typical characteristics of a benign lesion, a tissue biopsy is recommended. Conventional radiologic examinations are of limited value whereas histological and immunohistochemical confirmation is useful. The optimal treatment is wide excision with a sufficient surgical margin. In cases of DFSP of the breast, delayed reconstructive procedure can be considered since primary closure of the defect is not difficult due to sufficient soft tissue of the breast. Any concern about adequacy of complete excision, a large lesion with a negative margin, and a patient's severe anxiety about recurrence after excision can be considered as indications for delayed reconstruction (Figure 9). In addition, long-term postoperative follow up beyond several years is mandatory.

## Informed consent

Written informed consent was obtained from the patient for publication of this article and accompanying images. A copy of the written consent is available for review by the Editor-in-Chief of this journal.

## Competing interests

The authors declare that they have no competing interests.

## Authors' contributions

TH was responsible for the conception and design for the manuscript, the clinical work, the search for the literature, and the editing work. JK helped in the clinical work as well as the design for the manuscript. SW edited the manuscript and helped on the clinical work. CH provided overall supervision and contributed to concept. All authors read and approved the final manuscript.
